# Species Distribution Models and Impact Factor Growth in Environmental Journals: Methodological Fashion or the Attraction of Global Change Science

**DOI:** 10.1371/journal.pone.0111996

**Published:** 2014-11-11

**Authors:** Lluís Brotons

**Affiliations:** 1 CEMFOR-CTFC, Forest Sciences Center of Catalonia, Solsona, Catalonia, Spain; 2 CREAF, Centre for Ecological Research and Forestry Applications, Cerdanyola del Vallès, Catalonia, Spain; Consiglio Nazionale delle Ricerche (CNR), Italy

## Abstract

In this work, I evaluate the impact of species distribution models (SDMs) on the current status of environmental and ecological journals by asking the question to which degree development of SDMs in the literature is related to recent changes in the impact factors of ecological journals. The hypothesis evaluated states that research fronts are likely to attract research attention and potentially drive citation patterns, with journals concentrating papers related to the research front receiving more attention and benefiting from faster increases in their impact on the ecological literature. My results indicate a positive relationship between the number of SDM related articles published in a journal and its impact factor (IF) growth during the period 2000–09. However, the percentage of SDM related papers in a journal was strongly and positively associated with the percentage of papers on climate change and statistical issues. The results support the hypothesis that global change science has been critical in the development of SDMs and that interest in climate change research in particular, rather than the usage of SDM per se, appears as an important factor behind journal IF increases in ecology and environmental sciences. Finally, our results on SDM application in global change science support the view that scientific interest rather than methodological fashion appears to be the major driver of research attraction in the scientific literature.

## Introduction

Science is under continuous change and the appearance and development of new methodologies and approaches often has profound impact on the research panorama [1]. Species distribution models (SDM) exploring the association of environmental and species location data have rapidly developed over the last 15 years and appear to have had a great influence on environmental sciences and ecology in particular. SDM applications to climate change have been identified as the broadest research front in ecology and environment from Thomson ISI according to the clustering of the co-citing highly cited papers on this topic [2].

The popularity of SDM may be rooted in a range of different factors. Since understanding species distributions is a fundamental goal of ecology, the appearance of SDMs may have provided an efficient methodological approach to estimate species distributions and allowed the use of model outputs in a wide range of ecological applications (from species-energy relationships to niche conservationism [3]). The great availability of location data sets and environmental information in digital format (GIS) and the rapid development of statistical methods allowing efficient use of available information may have influenced the successful adoption of these techniques and their rapid spread in the ecological literature [3]. Being easy to implement using widely available GIS and distributional data coming from existing databases, SDMs may have benefited from a combination of fashion and ease of implementation. Alternatively, the popularity of SDMs may be related to the application of these techniques to expanding new ecological disciplines derived from an increasing interest in the effects of global change on biodiversity. SDMs allow a rapid estimation of the spatially explicit effects of drivers such as climate change on biodiversity at large spatial scales. Some seminal applications using SDMs have been instrumental in setting baselines of potential future impacts of climate change on a range of species [4].

In this work, I want to evaluate the impact of SDMs on the current status of ecological journals by asking the question to which degree of the use of SDMs in the literature is related to recent changes in the impact factors of ecological journals. The hypothesis evaluated derives from the idea that research fronts are likely to attract attention and drive research developments in a given discipline. Therefore, journals with a stronger focus on the research front should concentrate higher attention and receive more citations, thus benefiting from faster increases in their impact on the ecological literature. If this holds true, we predict that journals publishing more SDM-related articles should have benefited from the interest of this prolific field and show stronger increases in their citation rates and impact factors. However, if SDM usage is related to increases in citations rates, two main mechanisms may be identified as potential explanations to the observed patterns. First, SDM-related articles may be associated with studies on global change impacts on biodiversity (climate changes, land use changes and the impact of invasive species), and therefore, one should expect that the number of papers on these topics and not on SDM per se should better explain journal citation patterns. Alternatively, SDM may have influenced journal citations rates through of their intrinsic attraction as methodological novelty allowing the easy estimation of species distributions. In this case, I expect the number of papers on SDM to be associated to changes in the journal impact factor independently of the range of global change topics included in environmental and ecology journals.

## Methods

I used data from ISI web of science and test the prediction that the proportion of articles in a journal containing a larger number of SDMs related articles is related to the journal changes in impact factor during the period 2000–09. I used an objective method to select journals publishing a minimum number of articles related to SDM. This method included a general search for SDM related articles and the selection of a subset of articles included in non-multidisciplinary journals with more than 5 SDM articles published in the 2000–2009 period. Multidisciplinary journals were discarded because they included a much broader number of topics than thematic journals thus leading to potential biases in our blibliometric estimators. First, I searched the ISI web of science for articles containing the words “predictive species distribution model” “niche model” or “habitat suitability model” or a combination of these [3]. I identified a total of 2.118 articles leading to a total of 37.854 citations. Second, I selected a subset of articles published in currently active, non-multidisciplinary journals (according to the ISI categories, Thompson Scientific) with more than 5 SDM articles published during the period 2000–09 from ISI categories accounting for at least 2% of the total SDM references. These articles accounted for 1305 of the articles above. Although this subset, which accounts for over 60% of the SDM related articles included in our search, may not represent a comprehensive compilation of articles in the literature dealing with SDMs, I believe that due to the wide range of journals included, is representative of their distribution in the ecology, environmental sciences and biodiversity journals panorama. For this subset of journals mostly within the three subject areas mentioned above, I estimated impact factor trajectories and compiled the number of articles published per year during the period 2000–09. With the information derived from the databases, I was able to derive for, each of the 56 journals selected (Table S1), a measure of SDM relevance, *SDMr*, as the proportion of SDM related articles from the total number of articles published by the journal during the study period (range 0.01 to 12%). For each of the journals in this subset, I also obtained the number of articles published on different topics related to global change by searching for different combination of key words (“climate change*” (1505 articles), “land use change* or fragmentation” (603 articles) and “invasive species*” (1128 articles) in biology and ecology (“biolog* and ecology*”)) and calculated the proportion of articles for each topic in each journal (Table S1). Finally, I also used two additional different controls searches to account for general patterns in general ecological studies searching for the words “population and species” in biology and ecology (“biolog* and ecology*”) (2511 articles) and methodological biases searching for the word “statistics” in biology and ecology (“biolog* and ecology*”) (373 articles).

Impact factor (IF) is generally recognised as the primary measure of journal “quality” [6], but see [7]. Changes in the impact of the articles published in each journal (absolute increase) were quantified by calculating the slope of the regression of the journal's impact factor [5] and the respective year with positive slopes for journals with increasing impact factors and negative slopes for journals with decreasing impact. Finally, I also included, for each journal, the number of published articles during the study period and the year of the journal first issue (journal age) to account for general differences in article production and antiquity between journals [8]. I tested the role of journal descriptors on IF change and SDMr by means of linear models and forward variable selection using information theory based criteria (Bayesian Information Criteria, BIC) in R (package “MASS”, [9]). Both variables, IF change and SDMr, were log transformed to ensure normality. To deal with collinearity problems, I also used an analytical method named hierarchical partitioning (HP hereafter). HP reduces collinearity problems by determining the independent contribution of each explanatory variable to the response variable (I) and separates it from the joint contribution (J), resulting from correlation with other variables (for a detailed explanation of how HP works, see [10]. This allows ranking the importance of the covariates in explaining the response variable independently of the others covariates. Given its usefulness for complementing multiple regression analysis, I applied HP using the “hier.part package” in R [11].

## Results

**Figure 1:**
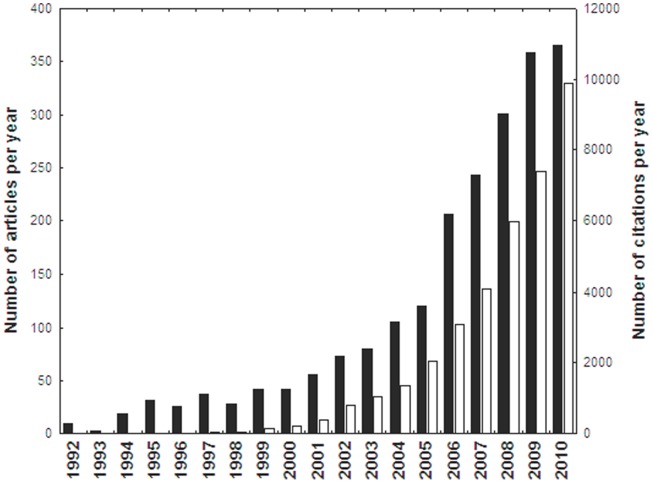
Number of published SDM related articles in the ecological literature (black bars) and number of citations received by these articles per year (white bars) during the period 1992–2010.

**Figure 2:**
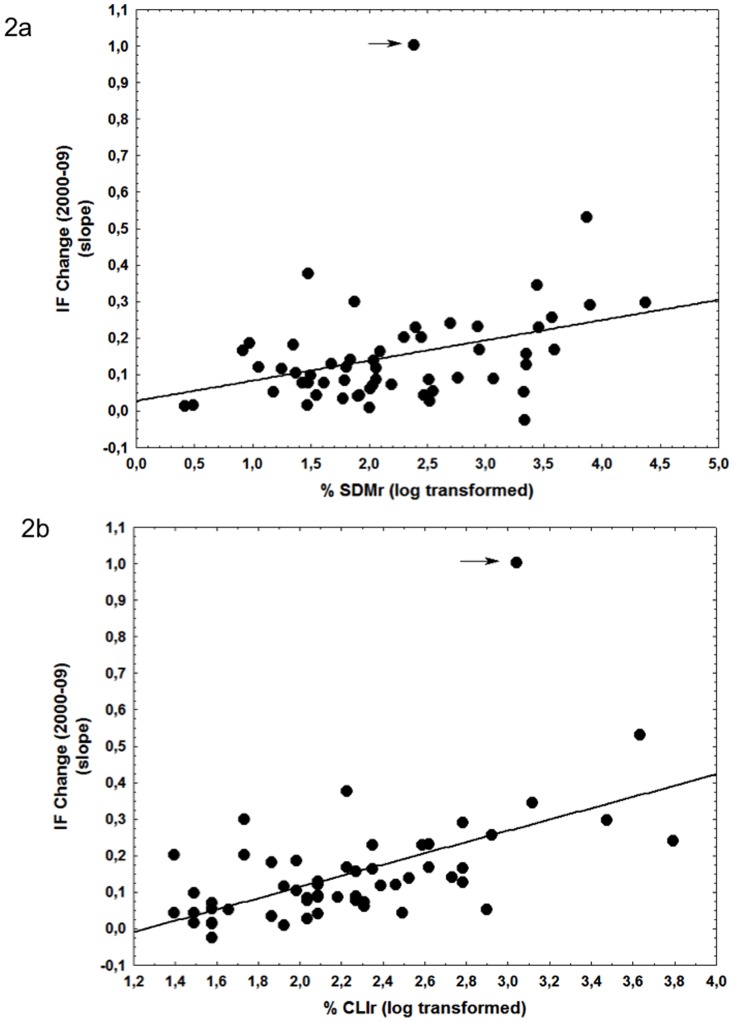
Increases in IF factors between 2000 and 2009 in relation to the percentage of SDM papers (SDMr, R^2^ = 0.12, *ß* = 10.68, *t* = 2.74, *d.f.* = 54, p<0.01) (a), and in relation to the percentage of climate change papers published in each journal (CLIr, R^2^ = 0.31, *ß* = 17.82, *t* = 4.98, *d.f.* = 54, p<0.001) (b). The arrow identifies the journal Ecology Letters.

The number of articles on SDM in the literature has rapidly increased during the period 2000–09 (Fig. 1) with the number of citations these papers are receiving also increasing rapidly with one third of the total amount of citations received in 2010. Ecology journals with a higher percentage of SDM related articles showed higher increases in their IFs between 2000 and 2009 (Fig. 2, Table S2). The total number of papers published by a journal, its IF at the beginning of the study period or the journal age were not significant factors behind changes in IF for the set of journals analysed and were thus discarded from the final model. The three ecology journals with values of SDMr larger than 5% (Diversity & Distributions, Global Ecology & Biogeography and Ecography) showed increases during the study period larger than 200% in their IF (Table S1). The relationship between changes in IF and SDMr was stronger and accounted for up to 16% of the variability when the journal Ecology Letters (experiencing a spectacular increase in IF during this period) was excluded from the analyses (*ß* = 11.05, *t* = 3.13 *d.f.* = 51, p<0.005).

SDMr was highly predictable from the combination of topics analysed and contained in a given journal. SDMr was in particular strongly and positively related to the number of articles on global change topics (climate change, *ß* = 5.53, *t* = 4.74, *d.f.* = 51, p<0.0001, Table S2) and to the percentage of articles on statistics published by a journal (*ß* = 29.69, *t* = 4.62, *d.f.* = 51, p<0.0001, Table S2). I also found a minor tendency for journals with lower IF in the year 2000 (*ß* = −0.00044, *t* = 2.44, *d.f.* = 51, p<0.05) and a lower number of total articles published (*ß* = −0.0042, *t* = 2.13, *d.f.* = 51, p<0.0001) to include a largest percentage of SDM related articles. The final model predicted 60% of variability in journal SDMr.

The effect of SDMr on IF change disappeared after the inclusion of variables accounting for the thematic scope of the journals. IF change was strongly and positively related to the number of climate change papers published (CLIr) in a journal during the study period (Fig. 2, Table S2). In fact, CLlr showed a much higher both, independent and joint explanatory power than any other variable included in the assessments, suggesting that it is a much stronger candidate to drive IF changes than SDMr or the others bibliometric descriptors used (Table S2).

## Discussion

My results indicate a positive relationship between the numbers of SDM related articles published in a journal and its IF increases during the period 2000–09. However, given the strong association between SDMr and the number of global change articles published in a journal, the role of SDM on IFs is likely to be an indirect effect of the increases in the journal IF being associated with a larger number of climate change articles published. The results support the hypothesis that global change science has been critical in the development of SDM and that climate change research in particular appears as an important factor behind increases of IF in ecological and environmental journals devoting larger attention to this topic.

Recent studies have found a positive trend in the number of articles cited by ecological journals in recent years leading to a potential for general increase in citation rates and thus impact factors [12], [13]. However, it seems that increases in IF are not evenly distributed with some journals getting a disproportionally larger share of the IF growth leading to changes in the potential impact of these journals [13], [14]. Our results indicate that journals with an overall higher percentage of SDMr, but specially those with more articles on climate change topics have grown at relatively faster rates than others [15]. However, SDM use in the journals included in the analyses did not directly drive IF increases in the set of journals analysed. The application of SDMs has been described as a one of the biggest emerging fronts in ecology in the last years [2], with a large number of highly impacting articles, and appears to be rapidly growing. SDM articles often use already existing and readily available environmental data and therefore are less constrained than more traditional ecology works based on field data thus opening the way to faster and more widespread publication on different issues of general interest to ecologists. Furthermore, available software makes SDM applications very easy and potentially articles using these methodologies may be easier to write than in other ecology areas. Other studies have indeed described increases in citations rates of ecological journals associated to the number of articles published on SDMs (i.e. invasion biology, [16]). However, our results do not support the view that journals publishing more SDM related articles receive more citations per se.

Rather, the role of SDM on IF trends disappeared when climate change was included in the analyses. This indicates that journals with a larger numbers of climate change related papers have indeed grown larger IFs in the 10-year period of our study. Journals with a higher proportion of SDMs also published more articles on hotter topics such as climate change or invasion biology [17] suggesting that the increase in impact factor is not a direct consequence of the number of SDM articles published. Overall, these results show that specific topics disproportionally drive changes in research attention and appeared to influence journal citation patterns [17]. The finding that climate change research contributes to the variability in recent IF increases of environmental and ecology journals supports the view that scientific interest and not methodological fashion appears to be a major driver of research attraction [18].

SDM usage appeared therefore related to changes in journal IF most likely because their development has been largely driven by applications in climate change science. SDM development has been instrumental in moving global change science forward due to the capability of the models to be used for a large number of species over large spatial scales [4], [16]. The real impact of SDM in ecology may be therefore be better interpreted as one of the foundation stones of global change science applications in ecology. SDMs have allowed the environmental research community to efficiently integrate the extensive availability of large-scale biological data, appropriate tools and environmental data sets into the growing needs of spatially explicit biodiversity assessments. SDMs may continue to play a significant role in the future panorama of ecology and environmental sciences as long as they remain as key methodological approaches in global change science. Spatial models allowing the projection of species distributions to future environmental conditions such as climate change are still required and tend to progressively become more complex to overcome the limitations of the correlative nature of SDMs [19]. However, I think that the challenges faced by model building in global change science will require flexible, integrative approaches allowing the use of extensively available data, and SDMs are likely to continue playing a significant role in this context.

## Supporting Information

Table S1 **Bibliometric information compiled on journals used in the analyses of the impact of species distribution modelling (SDM) on changes in the journals impact factor index (IF).**
(DOC)

Table S2 **Results of hierarchical partioning analyses showing the percentage of independent, joint and total explained variance for each considered variable.**
(DOC)
