# Supplementary material for: Species Distribution Models and Impact Factor Growth in Environmental Journals: Methodological Fashion or the Attraction of Global Change Science
Source: PLoS One. 2014 Nov 11;9(11):e111996. doi: 10.1371/journal.pone.0111996 (PMC4227683; doi:10.1371/journal.pone.0111996)
Supplement: Table S1 — Bibliometric information compiled on journals used in the analyses of the impact of species distribution modelling (SDM) on changes in the journals impact factor index (IF). (DOC) [file pone.0111996.s001.doc]

Table S1. Bibliometric information compiled on journals used in the analyses of the impact of species distribution modelling (SDM) on changes in the journals impact factor index (IF). SDMr. species distribution model related articles. Global change topics analysed (INVr. Invasion biology related articles; CLIr. climate change related articles; LANr. land use change and fragmentation related articles). Control topics included in the analses (POPr. articles on species and populations; STAr. articles on statistics). See methods for further detail.

| Journal | Year of journal first issue | Articles published (2000-09) | IF 2000 | IF change rate  (2000-09) | Percentage of articles on a given topic in the journal (2000-09) | | | | | | | | | | |
| --- | --- | --- | --- | --- | --- | --- | --- | --- | --- | --- | --- | --- | --- | --- | --- |
|  |  |  |  |  | SDMr | INVr | | CLIr | | LANr | | POPr | | STAr | |
| DIVERSITY AND DISTRIBUTIONS | 1998 | 568 | 2.109 | 0.297 | 0.123 | | 0.350 | | 0.070 | | 0.019 | | 0.083 | | 0.011 |
| ECOGRAPHY | 1978 | 832 | 1.683 | 0.291 | 0.061 | | 0.017 | | 0.030 | | 0.013 | | 0.059 | | 0.011 |
| GLOBAL ECOLOGY AND BIOGEOGRAPHY | 1991 | 571 | 1.026 | 0.532 | 0.060 | | 0.026 | | 0.084 | | 0.016 | | 0.033 | | 0.009 |
| LANDSCAPE ECOLOGY | 1987 | 797 | 1.408 | 0.167 | 0.045 | | 0.008 | | 0.024 | | 0.065 | | 0.041 | | 0.016 |
| RIVER RESEARCH AND APPLICATIONS | 1987 | 567 | 0.6 | 0.156 | 0.044 | | 0.004 | | 0.014 | | 0.005 | | 0.011 | | 0.000 |
| JOURNAL OF APPLIED ECOLOGY | 1964 | 1228 | 2.091 | 0.256 | 0.044 | | 0.029 | | 0.036 | | 0.018 | | 0.083 | | 0.011 |
| BIOLOGICAL CONSERVATION | 1968 | 2847 | 1.578 | 0.229 | 0.039 | | 0.015 | | 0.023 | | 0.027 | | 0.063 | | 0.003 |
| JOURNAL OF BIOGEOGRAPHY | 1974 | 1602 | 1.44 | 0.345 | 0.039 | | 0.013 | | 0.046 | | 0.002 | | 0.053 | | 0.007 |
| ECOLOGICAL MODELLING | 1975 | 3054 | 0.849 | 0.127 | 0.035 | | 0.005 | | 0.030 | | 0.011 | | 0.029 | | 0.009 |
| JOURNAL OF WILDLIFE MANAGEMENT | 1937 | 1777 | 1.436 | -0.024 | 0.035 | | 0.000 | | 0.002 | | 0.002 | | 0.024 | | 0.006 |
| ECOLOGICAL APPLICATIONS | 1991 | 1732 | 3.488 | 0.053 | 0.035 | | 0.036 | | 0.035 | | 0.029 | | 0.060 | | 0.009 |
| BIODIVERSITY AND CONSERVATION | 1992 | 1837 | 1.074 | 0.088 | 0.027 | | 0.011 | | 0.014 | | 0.014 | | 0.042 | | 0.002 |
| ANIMAL CONSERVATION | 1998 | 552 | 1.714 | 0.168 | 0.024 | | 0.004 | | 0.013 | | 0.007 | | 0.053 | | 0.007 |
| CONSERVATION BIOLOGY | 1987 | 2237 | 2.814 | 0.233 | 0.023 | | 0.018 | | 0.024 | | 0.016 | | 0.027 | | 0.005 |
| BIOLOGICAL INVASIONS | 1999 | 700 | 2.531 | 0.229 | 0.023 | | 0.284 | | 0.016 | | 0.004 | | 0.080 | | 0.004 |
| JOURNAL OF VEGETATION SCIENCE | 1990 | 965 | 1.589 | 0.091 | 0.020 | | 0.010 | | 0.010 | | 0.006 | | 0.009 | | 0.007 |
| GLOBAL CHANGE BIOLOGY | 1995 | 1624 | 3.775 | 0.242 | 0.018 | | 0.009 | | 0.100 | | 0.016 | | 0.009 | | 0.001 |
| WILDLIFE BIOLOGY | 1995 | 441 | 0.603 | 0.055 | 0.016 | | 0.000 | | 0.002 | | 0.005 | | 0.036 | | 0.002 |
| WILDLIFE RESEARCH | 1956 | 777 | 0.87 | 0.027 | 0.015 | | 0.009 | | 0.009 | | 0.006 | | 0.057 | | 0.001 |
| JOURNAL OF SEA RESEARCH | 1961 | 522 | 1.307 | 0.085 | 0.015 | | 0.000 | | 0.010 | | 0.000 | | 0.023 | | 0.000 |
| ACTA THERIOLOGICA | 1955 | 477 | 0.422 | 0.044 | 0.015 | | 0.002 | | 0.000 | | 0.000 | | 0.021 | | 0.006 |
| LANDSCAPE AND URBAN PLANNING | 1986 | 1112 | 0.637 | 0.202 | 0.014 | | 0.003 | | 0.004 | | 0.032 | | 0.014 | | 0.004 |
| ECOLOGY LETTERS | 1998 | 1258 | 1.88 | 1.003 | 0.014 | | 0.033 | | 0.042 | | 0.009 | | 0.025 | | 0.008 |
| SYSTEMATIC BIOLOGY | 1951 | 567 | 6.239 | 0.202 | 0.012 | | 0.000 | | 0.000 | | 0.000 | | 0.007 | | 0.005 |
| AQUATIC CONSERVATION-MARINE AND FRESHWATER ECOSYSTEMS | 1991 | 539 | 0.99 | 0.072 | 0.011 | | 0.015 | | 0.015 | | 0.004 | | 0.032 | | 0.002 |
| FRESHWATER BIOLOGY | 1971 | 1685 | 1.571 | 0.163 | 0.010 | | 0.015 | | 0.016 | | 0.005 | | 0.024 | | 0.004 |
| IBIS | 1859 | 927 | 1.077 | 0.087 | 0.010 | | 0.000 | | 0.012 | | 0.001 | | 0.025 | | 0.000 |
| RESTORATION ECOLOGY | 1993 | 721 | 1.024 | 0.119 | 0.010 | | 0.000 | | 0.017 | | 0.004 | | 0.025 | | 0.001 |
| JOURNAL OF ORNITHOLOGY | 1931 | 629 | 0.417 | 0.139 | 0.010 | | 0.000 | | 0.021 | | 0.000 | | 0.035 | | 0.002 |
| FOREST SCIENCE | 1954 | 633 | 0.966 | 0.072 | 0.009 | | 0.000 | | 0.002 | | 0.003 | | 0.003 | | 0.002 |
| ENVIRONMENTAL MANAGEMENT | 1976 | 1515 | 0.822 | 0.062 | 0.009 | | 0.006 | | 0.015 | | 0.010 | | 0.007 | | 0.005 |
| BIOLOGICAL JOURNAL OF THE LINNEAN SOCIETY | 1969 | 1528 | 2.316 | 0.009 | 0.009 | | 0.002 | | 0.007 | | 0.001 | | 0.075 | | 0.009 |
| FORESTRY CHRONICLE | 1922 | 830 | 0.417 | 0.043 | 0.008 | | 0.000 | | 0.020 | | 0.004 | | 0.002 | | 0.002 |
| CANADIAN JOURNAL OF FISHERIES AND AQUATIC SCIENCES | 1901 | 2172 | 1.685 | 0.040 | 0.008 | | 0.005 | | 0.010 | | 0.001 | | 0.006 | | 0.002 |
| ENVIRONMENTAL MODELLING & SOFTWARE | 1982 | 1117 | 0.545 | 0.299 | 0.008 | | 0.000 | | 0.004 | | 0.005 | | 0.003 | | 0.001 |
| ECOLOGY | 1920 | 3324 | 3.65 | 0.141 | 0.008 | | 0.022 | | 0.028 | | 0.006 | | 0.043 | | 0.007 |
| ECOLOGICAL RESEARCH | 1985 | 915 | 0.615 | 0.084 | 0.008 | | 0.024 | | 0.009 | | 0.004 | | 0.024 | | 0.001 |
| JOURNAL OF ANIMAL ECOLOGY | 1932 | 1198 | 2.862 | 0.121 | 0.008 | | 0.008 | | 0.019 | | 0.004 | | 0.051 | | 0.004 |
| CONDOR | 1898 | 959 | 1.207 | 0.035 | 0.007 | | 0.000 | | 0.006 | | 0.002 | | 0.017 | | 0.001 |
| FOREST ECOLOGY AND MANAGEMENT | 1976 | 4990 | 0.982 | 0.129 | 0.007 | | 0.003 | | 0.010 | | 0.005 | | 0.015 | | 0.002 |
| MARINE ECOLOGY-PROGRESS SERIES | 1979 | 5455 | 1.928 | 0.077 | 0.006 | | 0.007 | | 0.014 | | 0.001 | | 0.020 | | 0.001 |
| NORTH AMERICAN JOURNAL OF FISHERIES MANAGEMENT | 1988 | 1166 | 0.948 | 0.017 | 0.006 | | 0.001 | | 0.001 | | 0.001 | | 0.011 | | 0.003 |
| JOURNAL OF HERPETOLOGY | 1966 | 1026 | 0.562 | 0.044 | 0.006 | | 0.002 | | 0.001 | | 0.003 | | 0.019 | | 0.000 |
| JOURNAL OF MEDICAL ENTOMOLOGY | 1963 | 1623 | 1.051 | 0.098 | 0.006 | | 0.001 | | 0.001 | | 0.000 | | 0.009 | | 0.002 |
| AMERICAN NATURALIST | 1867 | 1653 | 3.944 | 0.077 | 0.005 | | 0.008 | | 0.014 | | 0.001 | | 0.029 | | 0.005 |
| MOLECULAR ECOLOGY | 1992 | 3492 | 2.769 | 0.377 | 0.005 | | 0.011 | | 0.013 | | 0.003 | | 0.053 | | 0.013 |
| ICES JOURNAL OF MARINE SCIENCE | 1943 | 1744 | 1.212 | 0.077 | 0.005 | | 0.001 | | 0.009 | | 0.000 | | 0.009 | | 0.003 |
| OIKOS | 1949 | 2453 | 2.461 | 0.105 | 0.005 | | 0.015 | | 0.008 | | 0.004 | | 0.030 | | 0.004 |
| EVOLUTION | 1926 | 2511 | 3.632 | 0.181 | 0.005 | | 0.003 | | 0.006 | | 0.000 | | 0.032 | | 0.005 |
| JOURNAL OF ARID ENVIRONMENTS | 1978 | 1614 | 0.664 | 0.116 | 0.004 | | 0.006 | | 0.007 | | 0.005 | | 0.015 | | 0.002 |
| CANADIAN JOURNAL OF ZOOLOGY-REVUE CANADIENNE DE ZOOLOGIE | 1922 | 1982 | 0.779 | 0.052 | 0.004 | | 0.000 | | 0.003 | | 0.002 | | 0.017 | | 0.001 |
| HYDROBIOLOGIA | 1948 | 5073 | 0.582 | 0.121 | 0.004 | | 0.003 | | 0.010 | | 0.001 | | 0.016 | | 0.002 |
| JOURNAL OF ENVIRONMENTAL MANAGEMENT | 1973 | 1830 | 0.61 | 0.186 | 0.003 | | 0.002 | | 0.008 | | 0.007 | | 0.007 | | 0.002 |
| PROCEEDINGS OF THE ROYAL SOCIETY B | 1800 | 2266 | 3.037 | 0.166 | 0.003 | | 0.013 | | 0.030 | | 0.002 | | 0.028 | | 0.002 |
| INTERNATIONAL JOURNAL OF REMOTE SENSING | 1977 | 3450 | 0.827 | 0.017 | 0.002 | | 0.000 | | 0.002 | | 0.002 | | 0.000 | | 0.000 |
| JOURNAL OF FISH BIOLOGY | 1969 | 3178 | 1.14 | 0.015 | 0.002 | | 0.002 | | 0.002 | | 0.000 | | 0.008 | | 0.001 |
