# Supplementary material for: Species Distribution Models and Impact Factor Growth in Environmental Journals: Methodological Fashion or the Attraction of Global Change Science
Source: PLoS One. 2014 Nov 11;9(11):e111996. doi: 10.1371/journal.pone.0111996 (PMC4227683; doi:10.1371/journal.pone.0111996)
Supplement: Table S2 — Results of hierarchical partioning analyses showing the percentage of independent, joint and total explained variance for each considered variable. (DOC) [file pone.0111996.s002.doc]

Table S2. Results of hierarchical partioning analyses showing the percentage of independent, joint and total explained variance for each considered variable when: (IF assessment 1) assessing the effects of general journal descriptors on changes in the journal impact factor index (IF). (IF assessment 2) assessing the effects of general and thematic, global change journal descriptors on changes in the journal impact factor index (IF). (SDM assessment) assessing the effects of general and thematic, global change journal descriptors on the percentage of SDM related articles. See methods for further detail.

| IF assessment 1 | | | |  | IF assessment 2 | | | SDM assessment | | | |
| --- | --- | --- | --- | --- | --- | --- | --- | --- | --- | --- | --- |
| Variables^1^ | Independent | Joint | Total^2^ |  | Independent | Joint | Total |  | Independent | Joint | Total |
| Antiquity | 5.31 | 0.94 | 6.25 |  | 3.09 | 3.16 | 6.25 |  | 4.08 | 8.89 | 12.98 |
| Articles published | 0.20 | 0.10 | 0.30 |  | 0.09 | 0.02 | 0.03 |  | **4.87** | **4.89** | **9.75** |
| IF 2000 | 6.7 | -0.80^3^ | 5.91 |  | 2.72 | 3.19 | 5.91 |  | **1.58** | **-1.40** | **0.02** |
| SDM_r_ | **9.3** | **2.91** | **12.22^4^** |  | 3.26 | 8.95 | 12.21 |  |  |  |  |
| INV_r_ |  |  |  |  | 1.98 | 5.34 | 7.73 |  | 10.46 | 16.66 | 27.12 |
| CLI_r_ |  |  |  |  | **19.58** | **11.89** | **31.47** |  | **16.14** | **19.33** | **35.47** |
| LAN_r_ |  |  |  |  | 2.33 | 6.67 | 9.00 |  | 10.86 | 19.12 | 29.98 |
| POP_r_ |  |  |  |  | 1.35 | 5.76 | 7.11 |  | 11.00 | 21.44 | 32.44 |
| STA_r_ |  |  |  |  | 4.38 | 9.50 | 13.88 |  | **11.86** | **21.51** | **33.37** |

^1^Antiquity (years from journal first issue), articles published (articles published in the journal during the period 2000-09), IF 2000 (impact factor of the journal the year 2000). Percentage of articles on a given topic in the journal (2000-09): SDMr (species distribution model related articles), INVr (Invasion biology related articles), CLIr (climate change related articles), LANr (land use change and fragmentation related articles), POPr (articles on species and populations), STAr (articles on statistics).

^2^ Total explained variance, correlation (in “R^2^”) of each of the variables included in the model with the response variable.

^3^ Negative joint variance indicates that the other variables act as suprressors of the particular variables.

^4^ Bold lettering indicates significant variables included in final models.
